# A reproducible picture of open access health facility data in Africa and R tools to support improvement

**DOI:** 10.12688/wellcomeopenres.16075.2

**Published:** 2021-02-16

**Authors:** Andy South, Ahmadou Dicko, Mark Herringer, Peter M. Macharia, Joseph Maina, Emelda A. Okiro, Robert W. Snow, Anelda van der Walt

**Affiliations:** 1 Department of Vector Biology, Liverpool School of Tropical Medicine, Liverpool, UK; 2 STATS4D, Dakar, Senegal; 3 The Global Healthsites Mapping Project, Amsterdam, The Netherlands; 4 Population Health Unit, Kenya Medical Research Institute - Wellcome Trust Research Programme, Nairobi, Kenya; 5 International Organization for Migration, Nairobi, Kenya; 6 Centre for Tropical Medicine and Global Health, Nuffield Department of Clinical Medicine, University of Oxford, Oxford, UK; 7 Talarify, Kleinmond, South Africa

**Keywords:** Open Data, Master Facility List, Africa, Health, COVID-19, R, GIS

## Abstract

**Background:** Open data on the locations and services provided by health facilities have, in some countries, allowed the development of software tools contributing to COVID-19 response. The UN and WHO encourage countries to make health facility location data open, to encourage use and improvement. We provide a summary of open access health facility location data in Africa using re-useable R code. We aim to support data analysts developing software tools to address COVID-19 response in individual countries. In Africa there are currently three main sources of such open data; 1) direct from national ministries of health, 2) a database for sub-Saharan Africa collated and published by a team from KEMRI-Wellcome Trust Research Programme and now hosted by WHO, and 3) The Global Healthsites Mapping Project in collaboration with OpenStreetMap.

**Methods:** We searched for and documented official national facility location data that were openly available. We developed re-useable open-source R code to summarise and visualise facility location data by country from the three sources. This re-useable code is used to provide a web user interface allowing data exploration through maps and plots of facility type.

**Results:** Out of 52 African countries, seven currently provide an official open facility list that can be downloaded and analysed reproducibly. Considering all three sources, there are over 185,000 health facility locations available for Africa. However, there are differences and overlaps between sources and a lack of data on capacities and service provision.

**Conclusions: **These summaries and software tools can be used to encourage greater use of existing health facility location data, incentivise further improvements in the provision of those data by national suppliers, and encourage collaboration within wider data communities. The tools are a part of the afrimapr project, actively developing R building blocks to facilitate the use of health data in Africa.

## Introduction

Evidence-based health planning and decision making requires information on the location of health facilities and the services they provide. This is the case both in the current epidemic and more routine times. When such health facility data are made openly available it allows them to be used by others and improved. The United Nations is calling for countries to disseminate health related data and incentivise use in the current COVID-19 pandemic: “National statistical offices need to focus on disseminating open data in a way that facilitates and incentivizes data use to contribute to the fight against the pandemic. National statistical offices should provide data on health resources and monitoring efforts”. In this paper we produce a reproducible picture of current open access data on health facility locations in Africa. We consider our main target audience to be data analysts in individual countries that wish to access and use health facility data to aid the response to COVID-19. The aim is that both the picture we produce and the re-usable software tools can contribute to that response.

The World Health Organisation (WHO) recommends the development and maintenance of a single authoritative geocoded master facility list (MFL) per country containing information about both public- and private-sector health facilities. Advice for the development, maintenance, and sharing of an MFL is provided in the comprehensive resource package ( WHO, 2018) which includes recommendations to share health facility data “as broadly as possible”. Several benefits of this sharing are identified, including that more users generate more value, increasing priority to government and therefore support; improved quality through detection of errors by increased users; and improving linkage and data exchange through consistency across information systems. Health facilities are constantly changing and lists are a challenge to maintain, leading to problems with completeness and timeliness. Health facility lists are also often fragmented and duplicated, hosted by various government departments, donor organisations, or other non-profit outfits and maintained as separate lists for sub-national regions ( Mpango & Nabukenya, 2020; Rose-Wood *et al.*, 2014). 

To address these difficulties the WHO HeRAMS (Health Resources and Services Availability Monitoring System) initiative “aims to ensure that core information on essential health resources and services is readily available to decision makers at country, regional and global levels”. It provides support to countries in the compilation, management, maintenance and dissemination of master facility lists (including mobile and temporary service facilities) and core information on operational and accessibility status, management and support, basic amenities, health information systems, health services and resources. The HeRAMS process ensures the data gathered is curated and validated by service providers themselves and promotes peer to peer and external verification mechanisms, leading to the production of agreed, authoritative information. HeRAMS is currently in operation in 12 African countries (Burkina Faso, Central Africa Republic, Chad, the Comoros, Ethiopia, Mali, Mozambique, Nigeria, Sudan, Somalia and Zimbabwe) and planned for 5 more (Benin, Cameroon, Niger, Mauritania and the Republic of the Congo). A HeRAMS-COVID19 module (in operation in Mali and Sudan) has been specifically designed to monitor health systems capacities and gaps in resources and services required for COVID19 case management. HeRAMS data are viewable through online dashboards at www.herams.org. The system does not provide open access to raw data so only the numbers of facilities are included in the analyses here. HeRAMS strongly advocates for open data, particularly on the health facility master list, but still supports temporary or partial restrictions by data owners and contributors (countries, NGOs) depending on the context.

In addition to the WHO resources there is an active community of practice, openHIE (Health Information Exchange), working on the collaborative development of health information system components for national governments. Resources created by WHO and OpenHIE are mostly targeted at data providers. In contrast, we are approaching the availability of health facility data from a data consumer perspective. What can data analysts do now to access, use, and potentially improve, health facility data to help respond to the COVID-19 pandemic? Our aim is not to create a new continent-wide database, instead we aim to make it easier for analysts to explore and improve existing sources, and to incentivise the provision of open data.

Health facility lists have proved to be a useful contribution during the early response to COVID-19 in some countries. For example, in Germany and the USA open data on health facility locations and capacity have been made available. These open data are being used by independent projects as a part of software tools designed to contribute to the epidemic response by mapping intensive care facilities (e.g. in USA and Germany) or travel time to testing sites ( Rader *et al.*, 2020). Open data on health facility capacities and bed occupancy released weekly by the US Department of Health and Human Services allowed the New York Times to create an interactive map showing how full individual hospital locations are. By combining open health facility data with other open datasets such as demographic data, it is possible to determine where additional testing facilities might be required, where hospitals may be overwhelmed, and where to send additional health care workers. In Kenya, open facility databases, travel times, bed capacity and health system surge capacity have been combined to aid in COVID-19 response ( Barasa *et al.*, 2020; Macharia *et al.*, 2020). Vaccine rollout planning requires health facility location data both for vaccination locations and to identify numbers of health workers who need to be prioritised. COVID-19 is not a problem that will be solved by governments alone, but needs a concerted effort with NGOs, private enterprises, start-ups, and citizens. Open data allows others to contribute.

More generally the availability and use of open data is a key element of the Principles for Digital Development that have been put together by the international development community to promote best practice in the use of digital tools in development programs. The nature of open licenses is a large topic. In this article we follow a general definition, from the Open Knowledge Foundation, that “Open means anyone can freely access, use, modify, and share for any purpose (subject, at most, to requirements that preserve provenance and openness)”. Free access, use and sharing are important to allow maximum benefit from the data.

**Figure 1.**
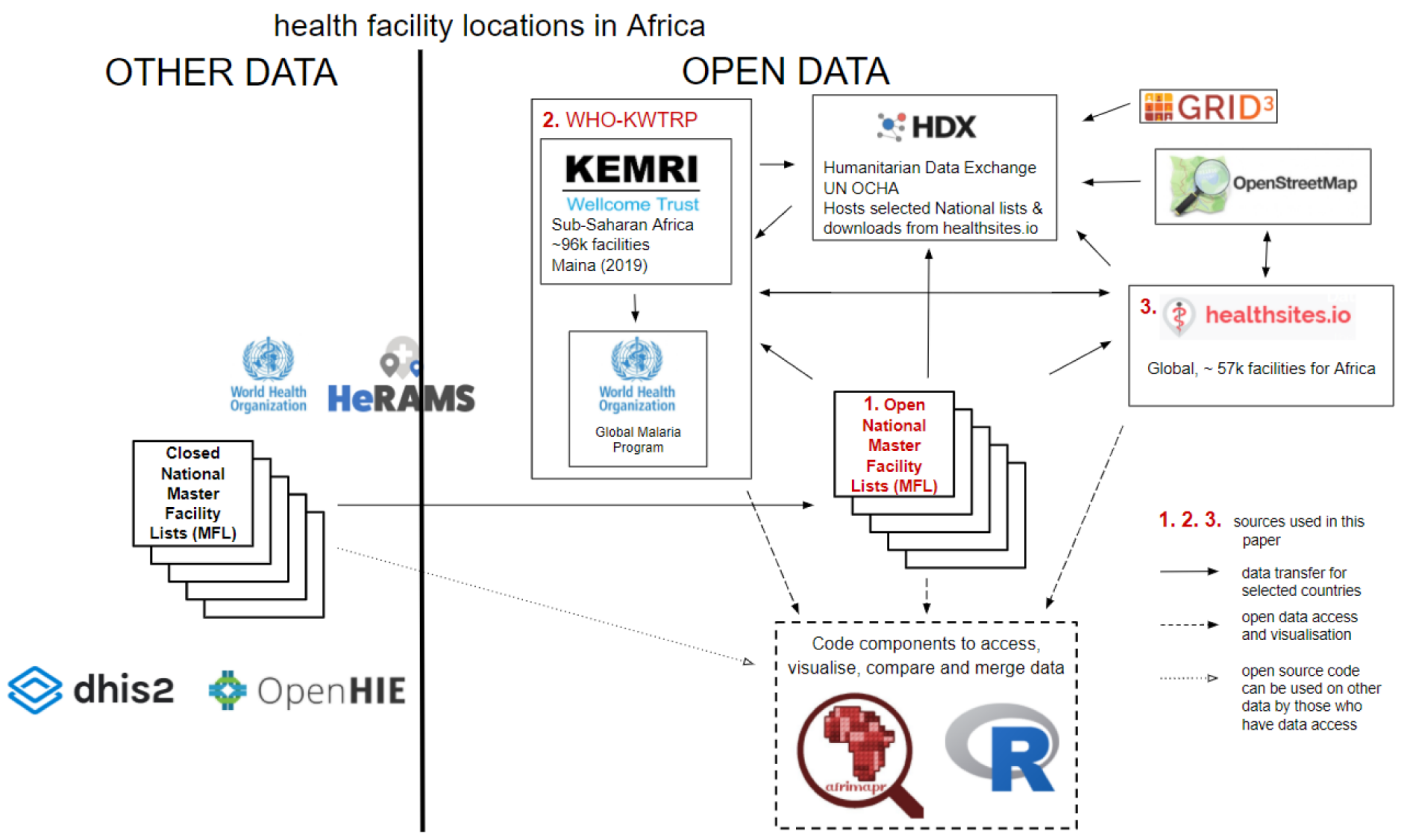
Main sources of open health facility location data for Africa and dataflows between them. The sources numbered 1–3 are summarised in this paper. Solid arrows indicate data transfers. Dashed arrows indicate data access and visualisation components created as a part of the afrimapr project and used in this paper. The dotted arrow at the lower left indicates that our open source components can be used, by in-country analysts, on closed data that we do not have access to.

Africa has three main, partially overlapping, sources of open data on health facility locations ( Figure 1). The first is available for a subset of countries and is not collated in one place, the second includes data for all sub-Saharan Africa and the third includes data for the whole continent. 

1. Hosted by country Ministries of Health or equivalent;
2. A collated dataset for sub-Saharan Africa published by researchers from KEMRI-Wellcome Trust Research Programme (KWTRP) in 2019 ( Maina *et al.*, 2019) and now hosted by the WHO Global Malaria Program(GMP); and
3. The Global Healthsites Mapping Project - healthsites.io is building an open data commons of health facility data with OpenStreetMap.

1. Direct from country Ministries of Health

In Africa, some countries provide open health facility lists, either as part of their official health information system (HIS) or as a separate open data asset. Many of the open health facility datasets for Africa have been developed through collaboration between Ministries of Health and donors (such as USAID, the European Union, and the Global Fund) or humanitarian aid organisations (such as the Red Cross), or even independent of Ministries of Health. The datasets vary considerably in terms of completeness, access method, data format, attributes available for each health facility (including geolocation), and facility types that are included.

2. Collated SSA facility database from WHO-KWTRP

In 2019 the Population Health Unit at KWTRP released a spatial database of health facilities managed by the public health sector in SSA ( Maina *et al.*, 2019). The article and an accompanying, behind the scenes piece ( Maina, 2019), describe the lengthy effort and difficulties in producing a continental-level dataset. The main effort took 6 years to complete, between 2012–2018, using multiple sources and location methods ( Maina *et al.*, 2019; Ouma *et al.*, 2018). Earlier, in 2003, the first ever MFL was developed for Kenya by KWTRP ( Noor *et al.*, 2003), and later updated in ( Noor *et al.*, 2009). The focus was on facilities that provide general medical care to the public, thus those that are exclusively private or only provide specialist services, such as oncology or dentistry, were excluded. These distinctions are not always easy to make. The 2019 dataset is now hosted by the WHO GMP ( WHO, 2019) with plans to refine and update. 

3. The Global Healthsites Mapping Project - healthsites.io

Healthsites is building an open data commons of health facility data with OpenStreetMap. The project leverages volunteered geographic information and the methods and infrastructure of OpenStreetMap to maintain baseline health facility data. Anyone can contribute locations and attribute data for individual sites by first creating an OpenStreetMap account. The project has a clear roadmap for how location coverage and accuracy can be improved over time by encouraging Ministries of Health to submit data, make them available to all within OpenStreetMap and initiate a process of checking and correction.

There is currently variable overlap between the three sources. Many coordinates in the WHO-KWTRP data were sourced from national ministries of health, some were sourced from healthsites.io. Some of the WHO-KWTRP data have subsequently been used to update healthsites.io. However, the WHO- KWTRP data cannot be directly added to healthsites.io or OpenStreetMap because they come from multiple sources and the licensing is not sufficient.

A fourth source, also potentially useful, is the Humanitarian Data Exchange (HDX). HDX is an open data sharing platform, provided by the United Nations Office for the Coordination of Humanitarian Affairs (OCHA). HDX hosts a wide range of humanitarian data, not just health facilities, on behalf of a variety of partners. We did not consider HDX explicitly here because in most cases the health facility data on HDX come from one of the other three sources, that we do consider. Health facility data from healthsites.io and OpenStreetMap is pushed to HDX monthly, and in some cases separate facility lists are also shared via NGO or national partners. For example, the GRID3 initiative has been working with local partners in the Democratic Republic of the Congo and has released health facility data for two provinces. Some national ministries of health have published their facility data on HDX, although it is now more common for them to do that direct from their own portals. Thus HDX often hosts several datasets for a single country. The WHO-KWTRP data indicates HDX as the main data source for nine countries ( Maina *et al.*, 2019), some of which are likely to have come from healthsites.io. In addition the WHO-KWTRP data have recently been made available in their entirety on HDX. We have made early investigations at accessing data from HDX through R using the rhdx package ( Dicko, 2020) and there is potential to extend that work.

Here we provide a reproducible summary of the three data sources and introduce software tools that we have developed to allow further investigation into how the data could be useful in the response to COVID-19. This analysis is a part of afrimapr, a new project actively developing R building blocks to facilitate the use of health data in Africa.

## Methods

Data were obtained from the three sources as follows.

### National data sources

Open health facility lists for all African countries were sought using a range of methods including web searches, Ministry of Health websites, links from the KWTRP collation ( Maina *et al.*, 2019) and open data portals. For countries where English is not the primary language, search terms were translated to French, Portuguese or Spanish.

Our criteria for including a country list in our analysis were:

I. clearly recognised by a country’s Ministry of Health as the official Master Facility List;
II. available for download (without the need to request permission); and
III. in a format that is easily machine readable for analysis (including Excel, CSV and JSON) but excluding PDF and other data embedded in reports.

Reproducible R code reading in and summarising the data, including a table of resources found by country, are available in this repository and accompanying report ( van der Walt & South, 2020b).

### WHO-KWTRP

The collated database was downloaded as a Microsoft Excel file from the WHO global Malaria Program where it is hosted ( WHO, 2019). The data have not changed since they were published. Re-usable R code to read and summarise these data are provided as a part of the afrihealthsites R package ( afrimapr, 2021). 

### healthsites.io

Data from healthsites.io can be queried via a Python API. This work prompted an update of the rhealthsites R package ( Dicko, 2020) to cope with recent changes to the Python API. rhealthsites allows R users to extract live data from the global healthsites database provided they have a current API key that can be obtained from healthsites.io after registering for an OpenStreetMap account (open to all). Code was written in the afrihealthsites R package to download healthsites data for Africa via the rhealthsites package and store the downloaded data. Thus, users of the afrihealthsites package can access either stored data for Africa (no account or key required), or live data (key required). The live data change over time as OpenStreetMap volunteers add and edit contributions. 

### Implementation

We used R (version > 3.5.0) and the following packages: mapview for interactive map plots, sf for manipulating geographic data, ggplot2 for facility type plots, patchwork for arranging plots, shiny for the web interface and rhealthsites for accessing data from healthsites,io ( Appelhans *et al.*, 2019; Chang *et al.*, 2020; Dicko, 2020; Pebesma, 2018; Pedersen, 2019; Wickham, 2009).

### Operation

The software we provide can be used in two ways by two potentially different communities. We provide:

1) a healthsites viewer web interface, that can be used by anyone with internet access
2) and an R package, afrihealthsites ( afrimapr, 2021), that can be used by those with knowledge of R to explore further.

**Figure 2.**
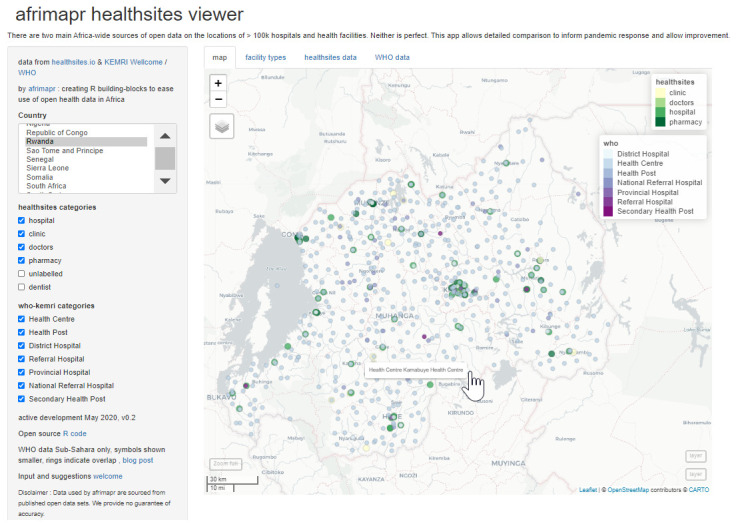
Screenshot from afrimap healthsites viewer showing health facility locations in Rwanda. There appear to be a wider geographic coverage of points from the WHO-KWTRP data (small blue-purple circles) than from healthsites.io (larger yellow-green circles). Users can zoom in to see facility locations in more detail.

**Figure 3.**
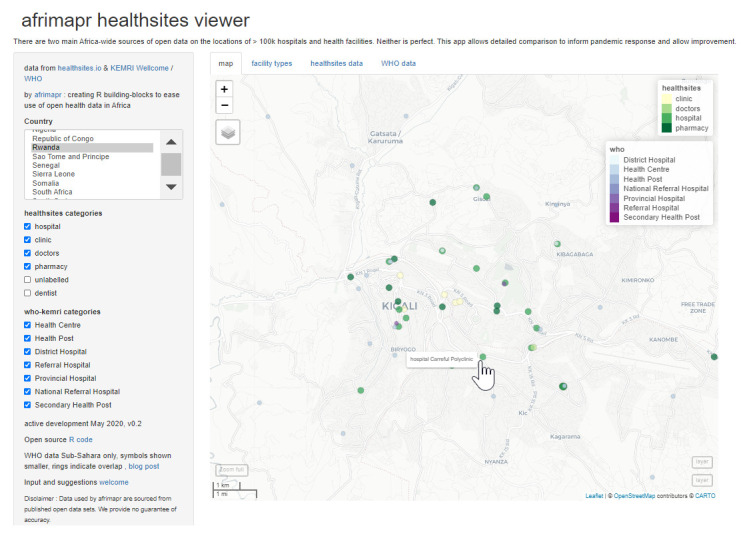
Screenshot from afrimapr healthsites viewer zoomed in on Kigali, the Rwandan capital. In large urban areas across the continent there tend to be more facilities in the healthsites.io dataset (yellow-green circles) than the WHO-KWTRP dataset (smaller blue-purple circles). This partly reflects there being different facility types in the two datasets.

The healthsites viewer is a web interface that allows users firstly to select options in a panel on the left that determines how the data are filtered and secondly select tabs on the right to view the filtered data in different ways ( Figure 2 and Figure 3). On the left, users can select one or more countries and tick or untick specific facility types from the two datasets; noting that the facility types available for the WHO-KWTRP dataset change according to the country selection. On the right users can select from tabs named ‘map’, ‘facility types’, ‘healthsites data’ and ‘WHO data’. The ‘map’ tab displays health facility locations on an interactive map that can be panned and zoomed. There is optional background mapping that displays e.g. place names and roads according to the zoom level. Hovering the cursor over a health facility will display its stored name. The ‘facility types’ tab displays two bar charts showing the frequency of the selected facility types; the upper chart for healthsites.io and the lower one for WHO-KWTRP. The ‘healthsites data’ and ‘WHO data’ tabs show spreadsheets of the selected raw data that can be searched and ordered to assist with detailed data exploration.

The afrihealthsites R package ( afrimapr, 2021) facilitates access to, and comparison between, health facility data for Africa from the WHO-KWTRP dataset, healthsites.io and national datasources. It requires R to run. The package contains internal documentation and is under active development so the code repository is the best place to seek usage instructions. The package contains functions called ‘afrihealthsites’ for accessing and visualising data from a single source, ‘facility_types’ for plotting the frequency of different facilities and ‘compare_hs_sources’ for comparing the location of facilities from two sources. Installation instructions are provided in the repository readme file. Documentation and examples for each function can be accessed by typing e.g. ?afrihealthsites or ?facility_types in the R console after installation. The code for the healthsites viewer is also provided within the R package, allowing R users to run it locally and modify for their own purposes.

## Results

The WHO-KWTRP dataset contains 98,745 facilities, with 96,395 geolocated while healthsites.io contains around 57,000 locations all spatially located.

Here we summarise three aspects of the different data sources.

A. The numbers of locations;
B. Classification of facility types (e.g. hospital, clinic, doctor); and
C. Attribute data useful for COVID response (e.g. capacity, number of beds, doctors, nurses etc.)

### A. Numbers of locations

**Figure 4.**
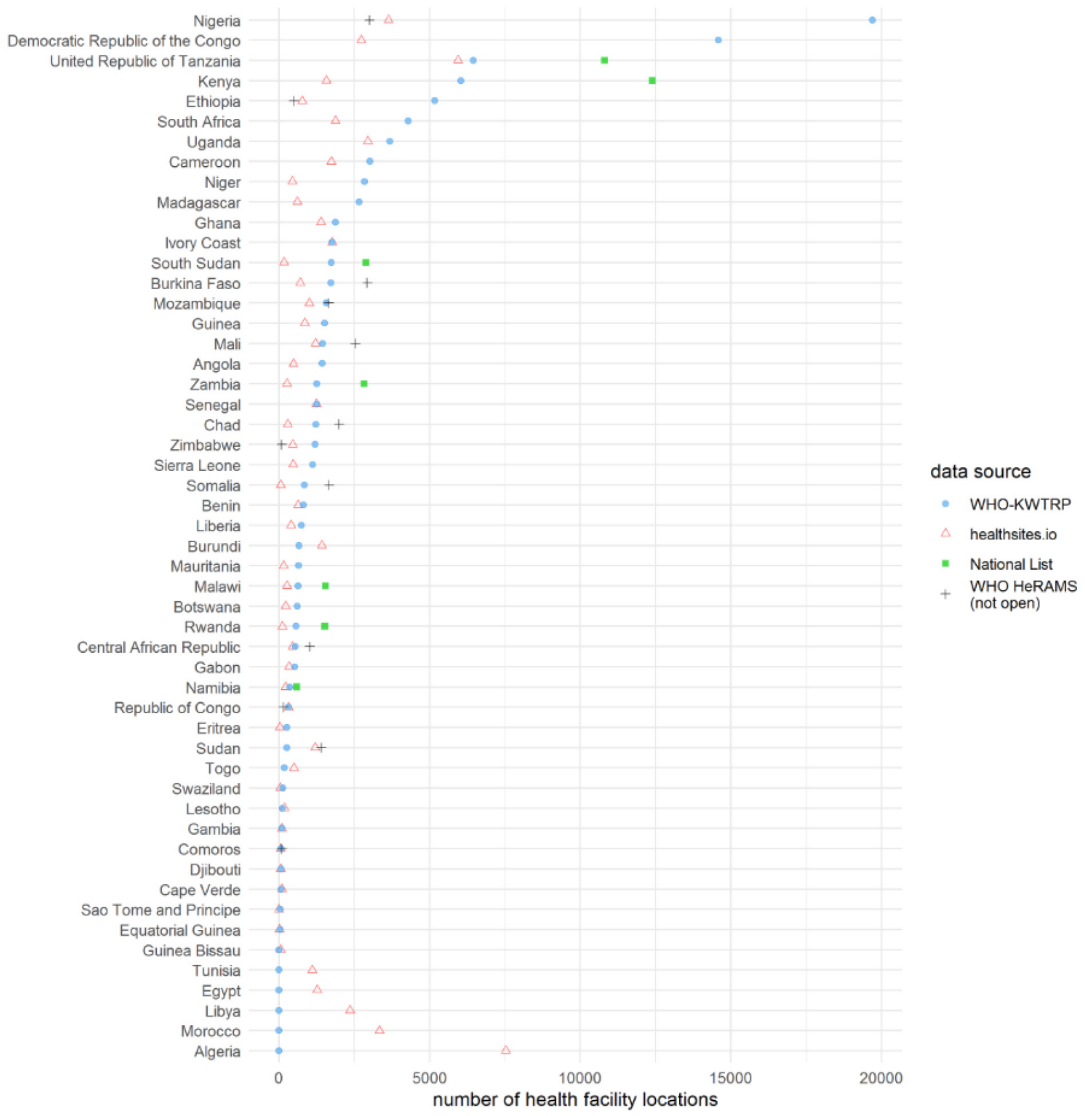
Numbers of health facility locations from three main open sources in Africa and WHO HeRAMS. WHO-KWTRP (blue circles) for sub-Saharan Africa, healthsites.io (red triangles) for all countries, and official National Master Facility Lists (MFL) available by machine-readable download for seven countries (green squares). WHO HeRAMS (black crosses) for 12 countries, can be viewed online but are not open.

National Master Facility Lists meeting our criteria outlined in the methods were found for seven African countries (Kenya, Malawi, Namibia, Rwanda, South Sudan, Tanzania, Zambia). These national sources contained more locations than for the corresponding country in either continent-wide dataset ( Figure 4).

Considering just the two continent-wide datasets, WHO-KWTRP contained more locations than healthsites.io for most countries, in many cases in excess of twice as many ( Figure 4). Exceptions to this included Burundi, Sudan, Togo and Lesotho where healthsites.io contained more locations, and Ivory Coast and Senegal where the numbers of locations were similar. In addition, the North African countries, Tunisia, Egypt, Libya and Morocco were not included in WHO-KWTRP so healthsites.io was the only available source.

The quantity of locations is, of course, a poor measure of the quality or completeness of the data sources and we will consider this in the discussion. Also, locations are of different types, as considered in the following section.

### B. Classification of facility types

**Figure 5.**
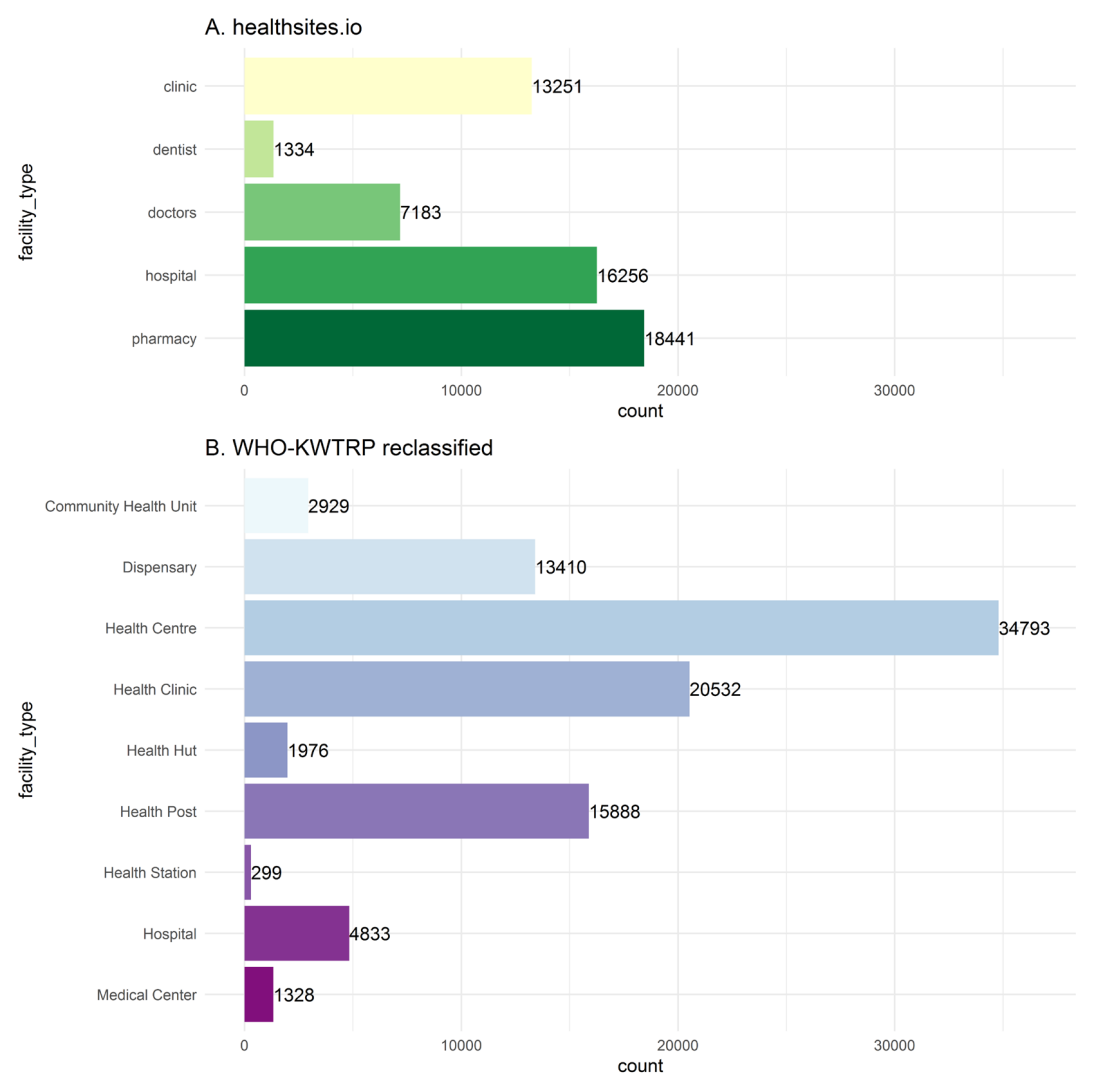
Health facility types in Africa from healthsites.io and reclassified to nine broad types from WHO-KWTRP. Frequency of health facility types in the two Africa-wide datasets. The data from WHO-KWTRP contained 172 different facility types, here they are reclassified to nine broad types following a similar method to ( Hulland *et al.*, 2019).

The WHO-KWTRP dataset deliberately retains facility type categories from the national data sources. As such it lists 172 facility types across the whole continent, partly a result of different classification systems and partly due to different languages, including Portuguese, French, Spanish and Arabic. To allow some summary and comparison ( Figure 5), we apply a reclassification similar to a nine-category classification used elsewhere ( Hulland *et al.*, 2019). healthsites.io facility types mostly fall into one of five categories: pharmacies, clinics, hospitals, doctors, and dentists ( Figure 5).

Our intention is to make clearer the differences in the data from the different sources, and the difficulties in comparing them. All of these classifications have some uncertainty associated with them that we consider in the discussion. However, to note broad patterns, the most common reclassified category in the WHO-KWTRP data is ‘Health Centre’ with more than 30,000 facilities and no clear equivalent in the healthsites.io data. There are about 16,000 facilities classed as ‘Hospital’ in the healthsites.io data and 5,000 in WHO-KWTRP. Dispensaries, which are likely to offer services as well as medicines (see discussion), number near 13,500 in WHO-KWTRP. Pharmacies, the closest class but excluded from WHO-KWTRP, number near 18,500 in healthsites.io. 

In general the country MFLs include a much broader list of facility types than both healthsites.io and WHO-KWTRP. For instance, the Kenya MFL includes facilities such as rehabilitation centres, nursing homes, blood transfusion centres, and more. The various types of facility lists also specify facilities at different levels of granularity, for example for Tanzania WHO-KWTRP lists no clinics *per se*, while the MFL specifies more than ten types of clinics, including dental clinic, eye clinic, and specialised polyclinic.

### C. Attribute data useful for COVID response (e.g. capacity, number of beds, doctors, nurses etc.)

The continent-wide data-sources have different attributes but do not contain useful amounts of information on capacities such as the numbers of beds or doctors. The healthsites.io data contains columns for staff_doctors, staff_nurses and beds, but has very few data in these columns. Less than 1000 facilities or 2% of the data currently contain these attributes. The WHO-KWTRP data does not contain any data on facility capacities. Of the seven national Master Facility Lists we included here, only the one for Kenya had information on facility capacities (but this list does not contain the coordinates of those facilities because whilst map locations can be viewed online the coordinates cannot be downloaded).

### Software tools for further data exploration

**Figure 6.**
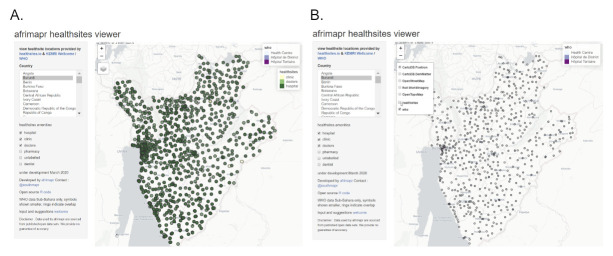
Burundi health facility locations in the afrimapr healthsites viewer show greater overlap between the two datasources. ( **A**) with the default view, of the two datasets enabled, most locations are indicated by a double ring indicating that facilities occur in both. ( **B**) turning off the healthsites.io layer reveals that the WHO-KWTRP data shows a very similar pattern.

Using the healthsites viewer available online, users can explore data from the two continent-wide datasets for individual countries in more detail. For example, looking at the map of Rwanda there appears to be a greater number of points from the WHO-KWTRP data (shown by the smaller blue-purple circles) than healthsites.io (shown by the larger yellow-green circles) ( Figure 2). This is supported by the summary statistics in Figure 4. In contrast, zooming in on the capital, Kigali, shows more records from healthsites.io ( Figure 3). For Burundi the situation is different, there appears to be a much greater overlap between the two datasets ( Figure 6), again this is supported by the summary of facility numbers in Figure 4.

**Figure 7.**
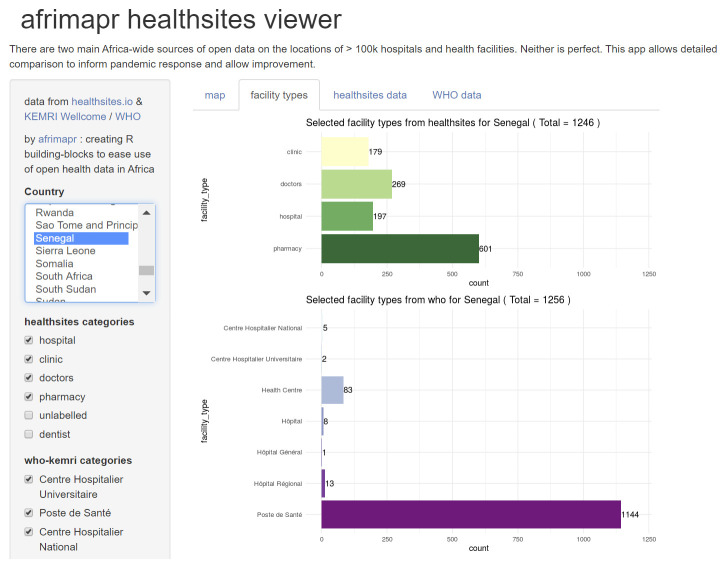
Screenshot from afrimapr healthsites viewer showing facility types for Senegal from healthsites.io and WHO-KWTRP.

Selecting the ‘facility type’ tab in the viewer can expose further differences between the continent-wide datasets. For example, for Senegal the numbers of locations in the two data sources are very similar, yet the distribution of facility types is very different ( Figure 7). A total of 50% of the healthsites.io locations are classed as pharmacies, whereas over 90% of the WHO-KWTRP locations are classed as Health posts.

Where the online viewer currently only displays the two continent-wide datasets, the afrihealthsites R package upon which it is built ( afrimapr, 2021) allows any other facility lists to be included in similar visualisations and comparisons. For example, the following R code in Box 1 will generate an interactive map ( Figure 8) comparing the locations of Zambian health facilities downloaded recently from the Ministry of Health, with those from WHO-KWTRP. The code is under active development, the exact syntax may change and the afrihealthsites repository ( afrimapr, 2021) should be consulted for current documentation and examples.

Box 1. **R code to compare two different facility lists and generate the map shown in Figure 8**

url_zambia <- "https://raw.githubusercontent.com/MOH-Zambia/MFL/master/geography/data/facility_list.csv"


dfzambia <- read.csv(url_zambia)


library(afrihealthsites)


# plot an interactive map of the locations from the two sources
compare_hs_sources('zambia',
        datasources = list('who', dfzambia),
        type_column = 'facility_type',
        label_column = 'name',
        lonlat_columns = c('longitude', 'latitude'))

**Figure 8.**
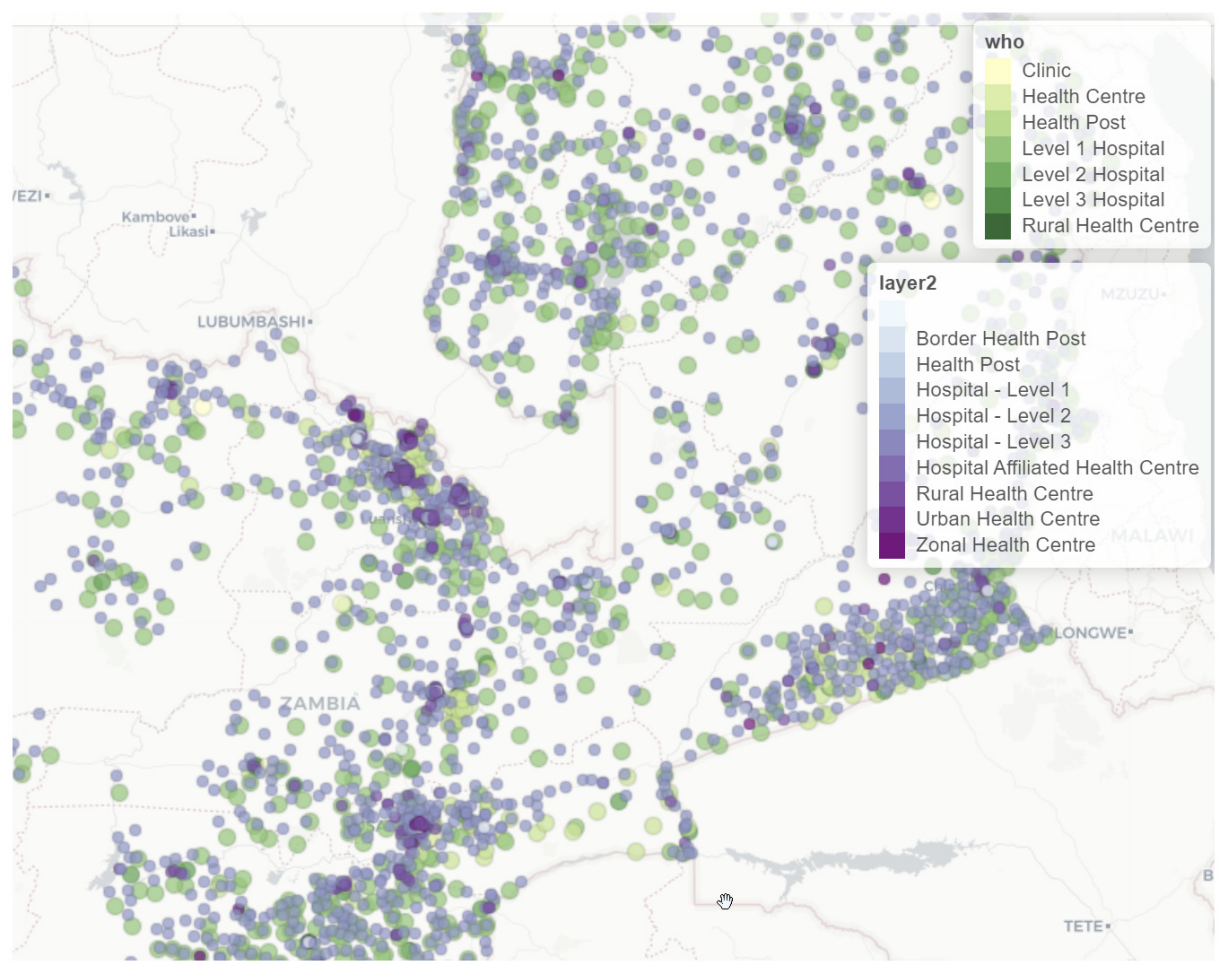
Comparison between Master Facility List for Zambia (layer2) and WHO-KWTRP data (who). Achieved with the afrihealthsites R code in Box 1. Shows that there are differences in the locations of facilities between the two datasources and in the facility types.

## Discussion

We have summarised the current state of open health facility location data in Africa using a reproducible analysis. This analysis shows both that a great deal has been done and that there is considerable room for improvement. From the perspective of data consumers, there are open data that could be useful in the response to COVID-19 but there are inadequacies and plenty of potential to improve data completeness to make them more useful. There are over 185,000 health facility locations available for Africa but information about services and infrastructure available at these facilities is scant.

Despite existing recommendations for health facility lists, it is non-trivial to obtain, analyse, and combine existing open data sets. Data formats, attribute names and content, and geolocation status vary greatly. Open health facility lists need to be combined with other open or proprietary datasets to address specific questions such as those being asked during the COVID-19 pandemic. What are the intensive care unit (ICU) capacity of hospitals in a specific area? How many respirators are available at health facilities in areas with high population density? Where is healthcare capacity likely to be exceeded? Knowing where facilities are located is a challenging first step but only becomes really useful if you know what services they provide.

We found just seven out of 52 African countries provide an open Master Facility List that can be downloaded and analysed relatively easily through a reproducible process ( van der Walt & South, 2020b). Table 1 provides a country by country summary of data availability including any current web links for download. A version of this table is also provided within the afrihealthsites R package along with functions to access it and we will update that as we become aware of changes. Our experience indicates that lists can exist for a country even when initial searches fail to find them, so our list of lists may not be comprehensive. The collation exercise that resulted in the WHO-KWTRP database found geocoded lists for 17 countries, and a further 11 where geocoding was absent, but many of these required either a login, manual extraction from pdfs or were not an official MFL (Table 1 in Maina *et al.*, 2019).

**Table 1.** Availability of official health master facility lists (MFL) or similar for African countries. Machine readable version provided in the afrihealthsites package where it can be queried and will be updated. After afrihealthsites installation the whole table will be returned by national_list_avail(), individual countries by e.g. national_list_avail(‘malawi’) and if data are available online this will return the link national_list_url(‘malawi’).

Country	iso3c	Official MFL accessible online	Data freely downloadable	Data machine readable	Data format	Downloaded data geocoded	MFL satisfies criteria for inclusion	Reason not included	Health facility data URL	About page URL	Alternative health facilities data source	Last updated	Owner	License	Comments
**Algeria**	DZA	no	yes	no	PDF	no	no	not machine readable	http://www.sante.gov.dz/media/ arisoft/pdfjs/web/viewer.html?file= http%3A%2F%2Fwww.sante.gov. dz%2Fimages%2Fpdf%2Fbulletins- officiels%2Fdoc%2Fetablissements -assurant-urgences.pdf	http://www.sante.gov.dz/ direction-generale-des- services-de-sante/305- etablissements-assurant- les-urgences.html		No date	Minsitry of Health		List of establishments providing emergency services from MoH- shows number of beds, services
**Angola**	AGO	no					no	not available							
**Benin**	BEN	no					no	not available							
**Botswana**	BWA	no	yes	no	PDF	no	no	not machine readable	http://168.167.134.123/en-gb/ Documents/Ministry%20of%20Lo cal%20Government%20and%20 Rural%20Development/MLGRD. Botswana%20Health%20Facilities. pdf	http://168.167.134.123/ en-gb/documents/ forms/allitems. aspx?rootfolder=/ en-gb/documents/ ministry+of+local+gove rnment+and+rural+dev elopment&folderctid=0 x01200000a87b983a65 ee4d9990244866d171 62&view=%7B62234187- 4a34-4517-b7fb- f84f2c89cd12%7D		2012	Ministry of Local Government and Rural Development		very limited information (updated 2012 - was valid until 2014)
**Burkina Faso**	BFA	no					no	not available							
**Burundi**	BDI	no					no	not available							
**Cameroon**	CMR	no	yes	no	PDF	no	no	not machine readable	https://www.dhis-minsante-cm.org/ portal/index.html	http://cis-minsante.cm/	https://data. ehealthafrica.org/ resource/f6281f48- f325-4f4a-8559-68a54 cc8a6ad?dataset=cam eroon-health-facilities	2016	Ministry of Health		Very low resolution PDF maps with limited detail - work funded through B&M Gates, CDC & WHO
**Cape Verde**	CPV	no					no	not available							
**Central** **African** **Republic**	CAF	no					no	not available							
**Chad**	TCD	no	no			no	no	not official MFL	https://data.ehealthafrica. org/resource/ac242db9-fd08-4ec4- ac13-2d0d39d89bf6?dataset=chad -health-facilities	https://data.ehealthafrica. org/resource/ac242db9- fd08-4ec4-ac13-2d0d3 9d89bf6?dataset=chad- health-facilities	https://data.humdata. org/dataset/chad-list- of-health-facilities- and-health-districts	2019	eHealth Africa		Hosted by eHealth Africa
**Comoros**	COM	no					no	not available							
**Democratic** **Republic of** **the Congo**	COD	no	no	yes	Excel	yes	no	not downloadable	https://front-office-resources. s3.eu-west-1.amazonaws.com/ usaid_ihp.xlsx	https://suivi-evaluation. ihp-prosani.com/		2019	Ministry of Health	CC-BY	Data not downloadable (error)
**Djibouti**	DJI	no					no	not available							
**Egypt**	EGY	no					no	not available							
**Equatorial** **Guinea**	GNQ	no					no	not available							
**Eritrea**	ERI	no					no	not available							
**Ethiopia**	ETH	no	yes	yes	SHP	yes	no	not official MFL	https://data.humdata.org/ dataset/679984dc-2e48-42a5- 9a2d-9135d8aad307/resource/ edc6b359-054f-4027-b1af- 2e8d34a0b28f/download/health_ facility.zip	https://data.humdata. org/dataset/ethiopia- health		2018	OCHA Ethiopia	Custom License	Data sourced from Ethiopian Ministry of Health, contributed by OCHA, hosted on HDX
**Gabon**	GAB	no	no			no	no	not downloadable	http://www.cnamgs.ga/anciensite/ localhost/wp/les-etablissements- agrees-par-la-cnamgs/hopitaux- cliniques-et-centres-de-soins- agrees-par-la-cnamgs/index.html			No date	National Health Insurance and Social Guarantee Fund		National Health Insurance and Social Guarantee Fund - list of approved hospitals
**Gambia**	GMB	no					no	not available							
**Ghana**	GHA	unclear	yes	yes	CSV	yes	no	not clearly labelled as official MFL	https://data.gov.gh/sites/default/ files/harvest_resources/HEALTH% 2520FACILITIES%2520IN%2520G HANA.csv	https://data.gov.gh/ dataset/health-facilities		2016	Ghana Open Data Initiative	Open Data Commons Open Database License (ODbL)	Part of the Ghana Open Data Initiative
**Guinea**	GIN	no	yes	yes	CSV	yes	no	not official MFL	https://data.humdata. org/dataset/300784da-ea33- 41a5-abd9-160915d33fb7/ resource/3d5e7ab6-5164-457d- bcc8-3cc1aa9b01aa/download/ structures_de_sante_guinee_vf.csv	https://data.humdata. org/dataset/structures_ de_sante_guinee_vf		2020	Ocha Guinea	CC0 (Public Domain)	Data sourced from Guinea Ministry of Health, contributed by OCHA, hosted on HDX
**Guinea** **Bissau**	GNB	no					no	not available							
**Ivory Coast**	CIV	no	yes	no	PDF	no	no	not machine readable	https://dipe.info/index.php/fr/ documentation/cartes-sanitaire/ send/3-carte-sanitaire/2-carte- sanitaire-de-la-cote-d-ivoire		https://data.humdata. org/dataset/health- facilities-2012	2008	Département Informatique et Santé		PDF in French - tables with health facility information found from page 56
**Kenya**	KEN	yes	yes	yes	Excel	no	yes	included	http://kmhfl.health.go.ke/#/facility_ filter/results	http://kmhfl.health. go.ke/#/home		No date	Ministry of Health		Full dataset is geocoded as a map visualisation is available, but downloaded spreadsheet does not contain coordinates
**Lesotho**	LSO	no					no	not available							
**Liberia**	LBR	no	yes	no	PDF	no	no	not machine readable	https://www.lisgis.net/pg_img/ Health%20Facilities%20by%20Cou nty%20and%20District.pdf	https://www.lisgis. net/page_info.php?&7d5f 44532cbfc489b8db9e12 e44eb820=MzY3	https://data.humdata. org/dataset/health- facilities-liberia-oct- 2014	2012	Liberia Institute of Statistics & Geo- Information Services		
**Libya**	LBY	no					no	not available							
**Madagascar**	MDG	no					no	not available							
**Malawi**	MWI	yes	yes	yes	Excel	yes	yes	included	http://zipatala.health.gov. mw/facilities	http://zipatala.health.gov. mw/about		No date	Ministry of Health		
**Mali**	MLI	no					no	not available							
**Mauritania**	MRT	no					no	not available							
**Morocco**	MAR	no					no	not available							
**Mozambique**	MOZ	yes	no	yes	Google Sheet	yes	no	permission needed to download	https://docs.google.com/ spreadsheets/d/1O8UUKnwH- XUZo7UJHVD-TpSm _7YQ3mJlqyQ8PqMcN84/pubhtml#	http://sis-ma.in/?page_ id=1085		No date	Ministry of Health		Google Sheet - needs permission to get access to original spreadsheet although (broken) link exists to download as ODS format
**Namibia**	NAM	yes	yes	yes	JSON	yes	yes	included	https://mfl.mhss.gov.na/api/ facilities.json	https://mfl.mhss.gov. na/pages/about		2020	Ministry of Health		Facility list in Excel provides access to basic data but API provides access to rich data
**Niger**	NER	no	no			no	no	not downloadable		https://data.ehealthafrica. org/resource/18ac9d25- 940e-4824-a468-a6ac1 d610b08?dataset=niger- republic-health-facilities		2019	eHealth Africa		
**Nigeria**	NGA	yes	no			Unsure (Have to request a key to download data but didn't receive one)	no	permission needed to download (request not answered)	https://hfr.health.gov.ng/download/ facilities	https://hfr.health.gov. ng/about-us	https://data. ehealthafrica.org/ resource/76506359- d2c5-4f12-b9b0- 4bee9dda2a33?dat aset=nigeria-health- care-facilities-primary- secondary-and-tertiary	2020	Ministry of Health		Facility list can be downloaded after completing a short form stating purpose of data download etc. Have tried twice but verification code for download isn't sent to email. Reached out to their contact email address but did not have a response.
**Republic of** **Congo**	COG	no					no	not available							
**Rwanda**	RWA	yes	yes	yes	CSV	yes	yes	included	https://moh.gov.rw/index. php?id=547	https://moh.gov.rw/index. php?id=547		No date	Ministry of Health		
**Sao Tome** **and Principe**	STP	no					no	not available							
**Senegal**	SEN	no					no	not available							
**Sierra Leone**	SLE	no	yes	yes	Excel	yes	no	not official MFL	https://data.humdata.org/ dataset/7453fb80-752b-4078-a892- d936f9846dab/resource/f78dc606- 04e2-4fb6-a7eb-9eb995c33f76/ download/1501-sierra-leone-health- centers.xlsx	https://data.humdata.org/ dataset/141121-sierra- leone-health-facilities		2015	Standby Task Force	Creative Commons Attribution for Intergovernmental Organisations	The list comes from a variety of MOH sources in the field and not from the MOH at national level. Collected by the Standby Task Force and hosted on HDX
**Somalia**	SOM	no	yes	yes	SHP	yes	no	not official MFL	https://data.humdata. org/dataset/b4fc93c9-8323- 4357-9f9f-5a22cdebca83/ resource/428bd8c0-8212-42c4- 922c-3c5f9a84ea20/download/ somalia-health-facilities.zip	https://data.humdata. org/dataset/somalia- health-facilities		2018	FAO SWALIM	CC0 (Public Domain)	Data from FAO SWALIM hosted on HDX
**South Africa**	ZAF	unclear	yes	yes	CSV	yes	no	not clearly labelled as official MFL	https://dd.dhmis.org/orgunits. html?file=NIDS%20Integrated&sour ce=nids&ver=1c11	https://dd.dhmis. org/index.html		2019	Ministry of Health		Download facility list by clicking on Download and selecting up to level 5
**South Sudan**	SSD	yes	yes	yes	CSV	yes	yes	included	https://www.southsudanhealth. info/facility/fac. php?list&s=0&p=0&ps=2889	https://www. southsudanhealth.info/		No date	Ministry of Health		
**Sudan**	SDN	no					no	not available							
**Eswatini**	SWZ	no					no	not available							
**Togo**	TGO	no					no	not available							
**Tunisia**	TUN	unclear	yes	yes	JSON	no	no	not clearly labelled as official MFL	http://fr-api.data.gov.tn/api/1.0/ dataset/586f67d4ea9cd/ search?from=0&size=10 http://fr-api.data.gov.tn/api/1.0/ dataset/586f6b401ce8b/ search?from=0&size=10 http://fr-api.data.gov.tn/api/1.0/dataset/586f605e167d2/search?from=0&size=10 http://fr-api.data.gov.tn/api/1.0/dataset/586f63acc2053/search?from=0&size=10 http://fr-api.data.gov.tn/api/1.0/ dataset/586f605e167d2/ search?from=0&size=10 dataset/586f63acc2053/ search?from=0&size=10	http://fr.data.gov.tn/27-a- propos.htm			Ministry of Health	Custom Open License	Four separate downloads: Centre de Soins de Santé de Base (Basic health care centre)Cabinet Medical Individuel (Individual medical office)Établissement de Santé Publique (Public health establishment)Hôpital de District (District hospital)
**Uganda**	UGA	yes	yes	no	PDF	no	no	not machine readable	https://health.go.ug/sites/default/ files/Signed%20n%20final%20mfl. pdf	https://www.health.go.ug/ cause/national-health- facility-master-list-2018/		2018	Ministry of Health		PDF report. Facility lists per region starts on page 24
**United** **Republic of** **Tanzania**	TZA	yes	yes	yes	Excel	yes	yes	included	http://hfrportal.moh.go.tz/index. php?r=facilities/exportToExcel& url=https%3A%2F%2Fresource map.instedd.org%2Fapi%2Fco llections%2F409.json%3Fpage %3Dall%26box%3D-180%2C- 90%2C179.99%2C90%26Admin_d iv%5Bunder%5D%3DTZ%26hum an%3Dtrue&report_title=List_of_ Facilities_with_Geo	http://hfrportal.moh. go.tz/index.php?r=page/ index&page_ name=about_page		2020	Ministry of Health		
**Western** **Sahara**	ESH	no					no	not available							
**Zambia**	ZMB	yes	yes	yes	CSV	yes	yes	included	https://raw.githubusercontent. com/MOH-Zambia/MFL/master/ geography/data/facility_list.csv	https://github.com/MOH- Zambia/MFL/		2019	Ministry of Health		Github repository with raw data
**Zimbabwe**	ZWE	no	no			No (full physical address is available)	no	not downloadable				No date	Health Professions Authority Zimbabwe		Quite complete list from health professions authority. not downloadable

Facility locations are useful as scientific data as well as for operations. The FAIR principles ( Wilkinson *et al.*, 2016) outline how the Findability, Accessibility, Interoperability, and Reusability of data can be improved to maximise reuse and value in science. They particularly promote that computers should be able automatically to find and use data. The healthsites.io health facility dataset is compliant with most of the principles. Table 1 shows how the country MFLs fared in terms of various indicators of FAIRness such as open licensing, machine readability, file formats, and metadata availability.

The quality of the data from the data sources we have considered is difficult to assess. The WHO-KWTRP data did go through an extensive quality control process ( Maina, 2019; Maina *et al.*, 2019), including both technical stages and comparison of approximate numbers of facilities with those indicated in national policies and strategic plans. Here we have made a briefer comparison between three sources and shown that there are various differences between them, resulting from differences in both intentions and methodologies. Without a more detailed country-by-country consideration it is not possible to say what proportion of facilities are covered, whether their coordinates put them in the right place and their recorded attributes are an accurate reflection of the services they currently provide. By making the data more accessible and enabling the comparison of different sources we hope to facilitate processes of quality assessment and improvement. 

In terms of numbers of facilities, we have shown that for the seven countries where we found open Master Facility Lists (MFLs) the numbers of locations in them were higher than in either of the corresponding continent-wide datasets. This is likely to be for a range of reasons. In some cases it appeared that the recent national MFLs contained a wider range of facility types than the WHO-KWTRP data. This tallies with the fact that the KWTRP collation process focussed on facilities providing general care, thus excluding private facilities and those offering specialist services. The KWTRP quality control process also identified that duplicates were a frequent issue and removed them ( Maina, 2019; Maina *et al.*, 2019). In this analysis we did not check the national MFLs for duplicate points so they could contribute to the higher numbers of locations.

Health facilities are changing all of the time, and that makes it difficult to keep an up-to-date list. Lower-order facilities get upgraded to include new functions, increased local investment leads to new facilities being built to support growing population needs, the health infrastructure is increasingly supported by community-level care providers often not captured in Master Facility Lists. The KWTRP collation process has not been updated since its completion in 2018, which will likely also contribute to differences with more recent national lists. The dynamic nature of the health system means that lists are unlikely to ever be entirely complete or accurate, but by making them open access the likelihood of updating with time is increased. The WHO HeRAMS system mentioned in the introduction seems to go a long way towards addressing these issues for the 12 countries it is currently used in, but it is difficult for us, as external analysts, to assess given that the data aren’t open. For most countries the WHO-KWTRP data contained more locations than healthsites.io, but for some major urban areas (e.g. Figure 3) healthsites.io seems to contain many locations not present in WHO-KWTRP. The generally higher number of points in WHO-KWTRP reflects a greater comprehensiveness given the closer link to the official sources. healthsites.io does encourage bulk import of data from official sources but currently relies more on crowd-sourced contributions from volunteers. The urban facility locations present in healthsites.io but not in the WHO-KWTRP data could be mostly private, for profit facilities that are generally not covered within the latter. The KWTRP data collation process focussed on public and other not-for-profit facilities. This was partly because the aim was to focus on facilities providing care for the general population and partly for pragmatic reasons around data availability. One exception to this case was Botswana, where private facilities were included because in that country they are more integrated into the government system and do provide care to the general public. Future efforts to improve the coverage of private facilities, both through official and crowd sources, would be beneficial.

Of course, even within the public and private sector, all facilities are not equal. The dots shown on our maps can represent major hospitals with hundreds of beds, health facilities with a handful of staff, pharmacies, and all points between. These differences are mostly stored in a single, imperfect character-string for each facility within a single column in the data tables. For the WHO-KWTRP data this column is called ‘Facility type’, for healthsites.io it is named ‘amenity’ - a reflection of the fact that it is derived from the amenity ‘tag’ used by OpenStreetMap volunteers to record the function of locations more generally (bar, post-office etc.). For the seven national MFLs that we obtained there were five different names for this (Facility type, Facility Type, facility_type, type, TYPE). These differences may seem trivial but they also provide hurdles for the unwary data analyst and have implications for data integration processes. 

There is no agreed, Africa-wide, quantitative classification of health facility types ( Ahmed *et al.*, 2015; Maina *et al.*, 2019). The WHO-KWTRP data retains the classification and type names used in the collated data sources. This has the advantage that facilities can be identified with respect to any national classification system where it exists. However, as a result, the continent-wide dataset has 172 different classes, making it somewhat difficult to manage. To gain some continent-wide overview of facility types, in a study of travel times to health facilities, ( Hulland *et al.*, 2019) reclassified these 172 classes into nine broader, English language, categories (hospital, health clinic, dispensary, community health unit, health post, health center, maternity ward, medical center, or polyclinic). We used a categorisation similar to this in our Figure 3. However, the difference between a health clinic, health centre or health post are not clear. National health facility type classifications do exist. The Kenyan Ministry of Health categorises their health services according to six defined levels ( GoK MoH, 2014). Broadly it contains, 1) Community (non facility based) services, 2) Dispensaries (pharmacy and health services but no inpatients), 3) Health centres (small hospitals with minimal facilities), 4) County hospital, 5) County referral hospital 6) National referral hospital. These between-country differences in how facilities are classified would be less important if lists contained information on capacities and services offered, however, that is seldom the case. 

The five main healthsites.io facility types (hospitals, clinics, doctors, pharmacies and dentists) have broader definitions still. Instructions are provided to volunteer OpenStreetMap mappers in a wiki. The instructions for hospital indicate “hospital is used for ... institutions for health care providing treatment by specialised staff and equipment, and typically providing nursing care for longer-term patient stays. In contrast, a medical centre with doctors for outpatient care only should be tagged amenity=clinic, and an individual doctor's office as amenity=doctors”. The instructions for ‘clinic’ indicate it should be applied to facilities with “10 or more of doctors, nurses and associated staff” and no inpatient admissions. The potential for it to be applied incorrectly to other specialised clinics is acknowledged. A pharmacy is defined as ‘a shop where a pharmacist sells medications’ where ‘dispensaries’ are defined in Kenya as having pharmacy and health services. Thus, with most facility types we find ourselves apparently trying to compare apples and oranges, aiming to make a useful combination of them.

A successful COVID-19 response requires access to health facilities across the spectrum with each facility type playing a specific role in providing counselling, testing, outpatient or inpatient care, medication, and so forth. Different facility types are likely to change in importance as the COVID-19 epidemic progresses. Being able to differentiate clearly between facility types will be useful. For epidemic surveillance the diagnostic abilities of facilities is of more interest. Facilities are currently being adapted to respond to the epidemic, therefore inevitably data even from a few months ago may be out of date. In this case data collection and provision as a part of facility modifications could help to ensure relevant data are available.

We found very few data on health facility capacities such as numbers of beds, doctors or nurses. The continent-wide datasources contained insufficient capacity attributes to be of much use. In the case of healthsites.io there is potential to hold capacity data but they have not been entered by volunteer mappers or gained from bulk imports. In the case of WHO-KWTRP, the data do not include services offered by each facility because most of the original sources did not contain such information. The one exception was the Kenyan MFL, available online, that does have information on the numbers of beds and cots per facility - but did not have coordinates that could be downloaded. HeRAMS does contain data on facility capacities that can be viewed online for the African countries it operates in, but these data cannot be downloaded without access rights.

Unique facility identification codes, where present, offer the potential to add data on facility capacities from other open data sources. Healthsites.io includes unique identifiers for each facility that are designed to allow linking with Master Facility lists or other OpenStreetMap data. Some of the national facility lists contain identifiers that allow linking to other national data. Facility identifiers were excluded from the WHO-KWTRP data because it would have been a huge exercise to check their validity and most of the original datasets did not contain them ( Maina *et al.*, 2019). Facility information data that could be used to augment MFLs include those from Service Provision Assessments (SPA), and Service Availability and Readiness Assessments (SARA) ( Sheffel *et al.*, 2018; WHO, 2015). The Service Provision Assessment (SPA), offered through the Demographic and Health Surveys Program, allows countries to gain a comprehensive view of health service delivery across health facilities. The survey evaluates topics such as infrastructure (e.g. water, electricity, infection control), resources (e.g. availability of vaccines, equipment and supplies for outpatient care), and services (e.g. curative services, emergency obstetric care, HIV testing services, laboratory diagnostic services). The sample of health facilities included in the SPA are normally selected from a country’s MFL. Survey data can be obtained from the DHS website and offer the potential to improve descriptive information available about the capacity of individual health facilities.

The lack of such capacity data for South Africa has prompted a group of researchers and volunteers to collate health facility and testing centre data ( Marivate & Combrink, 2020). Publicly available data from the Department of Health in South Africa ( NDOH, 2020) does not contain information about availability of beds, services or resources. Other open data sets are available that provide more descriptive information ( Dell, 2016). We are looking into ways to streamline the merging of such datasets via re-usable R code ( van der Walt & South, 2020a). There are existing tools from OpenHIE for detecting matches between two or more Master Facility Lists. However these are targeted at data producers rather than data consumers. They run within DHIS2 or independently but are not designed to be run on personal computers.

External observers may ask whether storage, analysis and visualisation of health facility data can be conducted within the country Health Management Information Systems (HMIS) such as DHIS2. HMIS, and DHIS2 specifically, are contributing to major improvement in the use of data in African public health, but much remains to be done ( Dehnavieh *et al.*, 2019; Maïga *et al.*, 2019). Few individuals within countries tend to have access to DHIS2 facility data and wider access would contribute to improvements in public health ( Maïga *et al.*, 2019). The WHO guidance for strengthening MFLs suggests that they are run independently ‘but integratable’ with other health information systems to allow easier changes in content or structure of either without disrupting the other ( WHO, 2018). For that reason, in South Africa a separate instance of DHIS2 from the main HMIS is used to store the list of facilities. From the perspective of external analysts the important part is that open data are made available and maintained (irrespective of how the HMIS and MFL are structured internally). Good open data are not just useful for NGOs, external analysts and the public, they also offer benefits for governmental staff. Open data make the exchange of data easier and more efficient within government organisations. When a government employee can freely access data from a public web link it makes them much more efficient than if they need to contact someone or gain access to a system that they may not have a password for.

When data are available, researchers tend to combine them with other data to generate new insights. This could be in both research and operational contexts. Since the publication of the WHO-KWTRP data ( Maina *et al.*, 2019) they have been used by other researchers to address local preparedness and response to viral hemorrhagic fevers (VHFs) ( Hulland *et al.*, 2019). In this process they combined the health facility data with estimates of suitability for different VHFs and spatial estimates of travel times to create maps identifying areas where risks and travel times are highest. More recently, maps of travel times to health facilities have been produced for the world ( Weiss *et al.*, 2020), and for older adults in sub Saharan Africa for the COVID-19 response ( Geldsetzer *et al.*, 2020). In both cases they used a combination of the WHO-KWRTP and OpenStreetMap/healthsites data covered here, with google maps added for the global analysis. Both acknowledged the uncertain quality of health facility location data and a comment piece on the latter paper suggested that formal investigation of data quality and duplication be included in future analyses of this type ( Hulland, 2020). Falchetta *et al.* (2020) use these data to estimate what would be needed at a continental scale to ensure universal access to healthcare according to common predefined targets. It includes potentially useful techniques for dealing with the lack of data on the services offered at facilities, e.g. to estimate bed capacities for individual hospitals by redistributing national statistics ( Falchetta *et al.*, 2020). There are undoubtedly other data that could usefully be combined with the locations of health facilities to inform the response to COVID-19 and other health issues in Africa. How can these health facility data and processes be improved? A key focus of the Healthsites project is the development of base line health facility data in OpenStreetMap in support of priority user stories and emergency hospital care. This Human Centered Design approach aims to improve data where it is needed most. Volunteer mappers and the infrastructures created by healthsites.io and OpenStreetMap offer considerable potential for data collection and validation particularly around the incorporation of official data from national ministries of health. A clear roadmap for data improvement has been created but requires funding for implementation. There are promising efforts using the data from WHO-KWTRP to indicate areas where OpenStreetMap health facility data are lacking ( HeiGIT, 2020a). Recent experience in India proves that official government health facility data can be successfully added to OpenStreetMap. Since April 2019 over 41,000 facilities have been added through bulk imports by the Indian OSM community including the commercial company RMSI ( HeiGIT, 2020b).

A progressive improvement in the capacities of ministries of health and national statistics agencies to deal with health data is occurring. National provision and maintenance of open data on health facility locations - including coordinates and details of services and infrastructure- can contribute to that improvement. Health data are a global good, and benefits will be achieved locally, regionally and globally. The wider community including, volunteer mappers, NGOs, academics, data-journalists and citizens, can be engaged to contribute and validate information. This would establish a collaborative, sharing approach where precious funding could be directed towards analysis and service improvement rather than data collection.

## Conclusion

We hope that this work will encourage greater use of existing health facility location data, partly for the benefits and partly to incentivise further improvements in the provision of those data. This can create a ‘virtuous cycle’ where data and uses improve together ( World Bank, 2019). The provision of facility locations, while demanding, is of limited use unless we know what services are provided at each location. We seek to encourage further collaborations between National data suppliers, wider data communities and NGOs (such as healthsites.io and OpenStreetMap) to improve data quality and use. The open software tools we are developing can contribute to these processes of use and improvement.

## Data availability

### Underlying data

All data underlying the results are available as part of the article and no additional source data are required.

### Extended data

**Code and data used in this article are available at:** https://github.com/afrimapr/afrihealthsites/blob/master/inst/rmd/2021-01-healthsites-paper-figs.Rmd

**Archived materials at time of publication:** http://doi.org/10.5281/zenodo.3871224 ( van der Walt & South, 2020b).

**License:** GNU Affero General Public License v3.0.

## Software availability

**The afrihealthsites healthsites viewer is available at:** https://andysouth.shinyapps.io/healthsites_viewer/.

**afrihealthsites source code available at:** https://github.com/afrimapr/afrihealthsites.

**Archived source code at time of publication:** https://doi.org/10.5281/zenodo.4469571 ( South & van der Walt, 2021).

**License:** GNU General Public License v3.0.
