# Supplementary material for: A reproducible picture of open access health facility data in Africa and R tools to support improvement
Source: Wellcome Open Res. 2021 Feb 16;5:157. Originally published 2020 Jul 6. [Version 2] doi: 10.12688/wellcomeopenres.16075.2 (PMC7780339; doi:10.12688/wellcomeopenres.16075.2)
Supplement: Peer review history

# Peer review: Reviewer response for version 2

**DOI:** 10.21956/wellcomeopenres.18296.r42619

**Published:** 2021-03-25

**Authors:** Olusesan Ayodeji Makinde

**Affiliations:** 1 Viable Knowledge Masters, Abuja, Nigeria

The authors have been responsive in addressing concerns raised on the earlier version of the manuscript. They have also improved the manuscript based on emerging evidence.

Is the work clearly and accurately presented and does it cite the current literature?

Yes

If applicable, is the statistical analysis and its interpretation appropriate?

Not applicable

Are all the source data underlying the results available to ensure full reproducibility?

Yes

Is the study design appropriate and is the work technically sound?

Partly

Are the conclusions drawn adequately supported by the results?

Partly

Are sufficient details of methods and analysis provided to allow replication by others?

Partly

Reviewer Expertise:

Epidemiology, Health Facility Assessments, Health Informatics

I confirm that I have read this submission and believe that I have an appropriate level of expertise to confirm that it is of an acceptable scientific standard.

# Peer review: Reviewer response for version 2

**DOI:** 10.21956/wellcomeopenres.18296.r42622

**Published:** 2021-02-16

**Authors:** Caleb Kipkurui Kibet

**Affiliations:** 1 International Center of Insect Physiology and Ecology, Nairobi, Kenya; 2 OpenScienceKE, Nairobi, Kenya

The authors have satisfactorily addressed my comments.

Is the work clearly and accurately presented and does it cite the current literature?

Yes

If applicable, is the statistical analysis and its interpretation appropriate?

Not applicable

Are all the source data underlying the results available to ensure full reproducibility?

Yes

Is the study design appropriate and is the work technically sound?

Yes

Are the conclusions drawn adequately supported by the results?

Yes

Are sufficient details of methods and analysis provided to allow replication by others?

Partly

Reviewer Expertise:

Open Science, Research Data Management, Bioinformatics

I confirm that I have read this submission and believe that I have an appropriate level of expertise to confirm that it is of an acceptable scientific standard.

# Peer review: Reviewer response for version 1

**DOI:** 10.21956/wellcomeopenres.17637.r41741

**Published:** 2020-12-24

**Authors:** Caleb Kipkurui Kibet

**Affiliations:** 1 International Center of Insect Physiology and Ecology, Nairobi, Kenya; 2 OpenScienceKE, Nairobi, Kenya

The paper by Andy *et al.,* 2020, "A rapid and reproducible picture of open access health facility data in Africa to support the COVID-19 response,"  provides a useful open tool for visualizing open Africa health facility data sourced from three sources: WHO-KWTRP, Healthsites.io, and National data source. They develop a shinny app to allow users to interact with the data: filter and visualize. Further, the authors provide an R-package that can be used to explore the data by users familiar with R, thereby catering to the needs of varied users of their resource. The shiny app visualizes the data from only two sources and does not include the data you sourced from the Master Facility Lists. With a locally installed package, the user can download Facility data for comparison, as demonstrated in Box 1.

 Therefore, this study's main contribution is the ability to visualize existing data and compare data from various sources. However, the highlighted usage for COViD-19 is not supported by the data they have, neither have they used it as a case study. Besides, they state that "The continent-wide data-sources have different attributes but do not contain useful amounts of information on capacities such as the numbers of beds or doctors." Kindly mention it as one of the potential uses and remove it from the title.

 I would have loved a discussion using the FAIR  principles( https://www.force11.org/fairprinciples) on how the data compare, especially as you highlighted the lack of interoperability of the data sources and provide some recommendations for achieving that.

 Other than the table in the paper, how can the users access a list of facility lists that pass your criteria? How can the users access detailed documentation on the tool?

Is the work clearly and accurately presented and does it cite the current literature?

Yes

If applicable, is the statistical analysis and its interpretation appropriate?

Not applicable

Are all the source data underlying the results available to ensure full reproducibility?

Yes

Is the study design appropriate and is the work technically sound?

Yes

Are the conclusions drawn adequately supported by the results?

Yes

Are sufficient details of methods and analysis provided to allow replication by others?

Partly

Reviewer Expertise:

Open Science, Research Data Management, Bioinformatics

I confirm that I have read this submission and believe that I have an appropriate level of expertise to confirm that it is of an acceptable scientific standard, however I have significant reservations, as outlined above.

# Response

**Published:** 2021-01-27

**Authors:** Andy South

Thank you Caleb for your time and consideration.

 Reviewers points in italics, our responses marked R., added text in bold

*However, the highlighted usage for COViD-19 is not supported by the data they have, neither have they used it as a case study. Besides, they state that "The continent-wide data-sources have different attributes but do not contain useful amounts of information on capacities such as the numbers of beds or doctors." Kindly mention it as one of the potential uses and remove it from the title.*

 R. We have removed COVID-19 from the title as requested.

*I would have loved a discussion using the FAIR  principles (https://www.force11.org/fairprinciples) on how the data compare, especially as you highlighted the lack of interoperability of the data sources and provide some recommendations for achieving that.*

 R. Good idea we have added brief discussion of FAIR principles to the discussion :

**“Facility locations are useful as scientific data as well as for operations. The FAIR principles (Wilkinson et al., 2016) outline how the Findability, Accessibility, Interoperability, and Reusability of data can be improved to maximise reuse and value in science. They particularly promote that computers should be able to automatically find and use data. The healthsites.io health facility dataset is compliant with most of the principles. Table 1 shows how the country MFLs fared in terms of various indicators of FAIRness such as open licensing, machine readability, file formats, and metadata availability.”**

*Other than the table in the paper, how can the users access a list of facility lists that pass your criteria? How can the users access detailed documentation on the tool?*

 R. Good points. We have added the national facility list table to the afrihealthsites package and will maintain it on Github. We have added this to the legend to Table 1 :

**Machine readable version provided in the afrihealthsites package where it can be queried and will be updated. After afrihealthsites installation the whole table will be returned by national_list_avail(), individual countries by national_list_avail(‘malawi’).**

 Information about documentation is provided in the Operation section at the end of the Methods. We added this sentence : **“Installation instructions are provided in the repository readme file. Documentation and examples for each function can be accessed by typing e.g. ?afrihealthsites or ?facility_types in the R console after installation.”**

# Peer review: Reviewer response for version 1

**DOI:** 10.21956/wellcomeopenres.17637.r41445

**Published:** 2020-12-11

**Authors:** Doreen Wamiti

**Affiliations:** 1 Population Services International, Nairobi, Kenya

The work is accurately stating the available MFLs and also clearly showcasing how all the available facility locations complement each other.

 This work is also highlighting potential opportunities for improvement of the facility types, country MFLs, and geolocations. The sources used to inform this work are credible sources and probably there could be more sources that can be looked into.

 The fact that this work is able to highlight additional useful information like service provision and capacity which is missing, presents opportunities for other researchers to gather more information on this and also for the facility lists to be improved.

Is the work clearly and accurately presented and does it cite the current literature?

Yes

If applicable, is the statistical analysis and its interpretation appropriate?

I cannot comment. A qualified statistician is required.

Are all the source data underlying the results available to ensure full reproducibility?

Yes

Is the study design appropriate and is the work technically sound?

Yes

Are the conclusions drawn adequately supported by the results?

Yes

Are sufficient details of methods and analysis provided to allow replication by others?

Yes

Reviewer Expertise:

Maternal and child health

I confirm that I have read this submission and believe that I have an appropriate level of expertise to confirm that it is of an acceptable scientific standard.

# Response

**Published:** 2021-01-27

**Authors:** Andy South

Thank you Doreen for your time and consideration.

*Reviewers points in italics*, our responses marked R.

*The sources used to inform this work are credible sources and probably there could be more sources that can be looked into.*

 R. Good suggestion. We have added mention of other sources to Figure 1 and added text and to Figure 4 about the WHO initiative HeRAMS.

# Peer review: Reviewer response for version 1

**DOI:** 10.21956/wellcomeopenres.17637.r39729

**Published:** 2020-09-23

**Authors:** Chigozie Edson Utazi

**Affiliations:** 1 WorldPop Research Group, School of Geography and Environmental Science, University of Southampton, Southampton, UK

The paper addresses an important challenge in health service provision and delivery in Africa by creating an open database of health facilities across the continent from multiple data sources. The aim of the project is to provide end users/health practitioners with much needed data to support Covid-19 response in these countries. There are, however, non-ignorable flaws in the paper that jeopardize this aim. The data that have been assembled are not of good quality and do not contain sufficient information to enable its use for Covid-19 response in many countries as the authors have noted. More detailed comments on the paper are provide below.

1. Any endeavour to develop a continent-wide master facility list without close collaboration with governments is likely to flounder. One, many health policies are implemented through governments and where these governments do not accept an externally-developed database such as the one created in this paper, it’s use and impact will be greatly limited. The authors mentioned that the database was created by assembling data from multiple sources including publicly-available national master facility lists, but there is no mention of collaboration with ministries of health, which is vital to guarantee the quality and acceptability of the data. 
2. Classification of health facilities varies from country to country. One would expect that an effort of this magnitude would facilitate a more generally acceptable classification scheme. This inconsistency in the data makes it even more challenging to use for multi-country studies. In some countries, as the authors mentioned, this list includes pharmacies and all kinds of clinics. These facility types are usually privately-owned and it is the case that they are opened and closed irregularly. Often, such facilities do not offer basic health services to be worthy of inclusion in any quality health facility database. I think users will be more interested in a more standardized database. 
3. There is no clear plan or mechanism for updating and validating the database. As the authors rightly pointed out, health facilities change all the time. The WHO-KWTRP has not been updated since creation in 2018. Data from healthsites.io are mainly updated through OpenStreetMap volunteers, with all the challenges that are inherent in such an ad hoc process. My take is that there is no meaningful validation of health facilities that can be done without the support and participation of governments. This is a major dent on the credibility of the database.  
4. Most importantly, the authors claim that the database is primarily geared towards facilitating Covid-19 response. If most of the facilities in the database have missing attributes, it will be extremely difficult to assess their utility for Covid-19 intervention. This calls the rationale behind the paper into question.

Is the work clearly and accurately presented and does it cite the current literature?

Yes

If applicable, is the statistical analysis and its interpretation appropriate?

Not applicable

Are all the source data underlying the results available to ensure full reproducibility?

Yes

Is the study design appropriate and is the work technically sound?

Partly

Are the conclusions drawn adequately supported by the results?

Partly

Are sufficient details of methods and analysis provided to allow replication by others?

Partly

Reviewer Expertise:

Spatial and spatiotemporal statistics; child health

I confirm that I have read this submission and believe that I have an appropriate level of expertise to confirm that it is of an acceptable scientific standard, however I have significant reservations, as outlined above.

# Response

**Published:** 2020-10-27

**Authors:** Andy South

We thank the reviewer for their time and useful insights. We had been waiting for further promised reviews but are responding now given the delay. We will amend the manuscript after they are received. 

We would like to correct a misconception. The reviewer suggests that our main aim is to create an open database of health facility locations across Africa and points out problems that jeopardise that aim. Our main aim was not to create a continent-wide database. We will add some text to explicitly state that. We already indicate in the title that our main aim is a “reproducible picture of open access health facility data in Africa”, and in the first paragraph of the abstract we say : “We provide a summary of open access health facility location data in Africa using re-useable code. We aim to support data analysts developing software tools to address COVID-19 response in individual countries.” In the final paragraph of the abstract: “We suggest that these summaries and tools will encourage greater use of existing health facility location data, incentivise further improvements in the provision of those data by national suppliers, and encourage collaboration within wider data communities.”

We agree completely with the reviewer about the difficulties of developing a continent-wide database which is why our aim is instead to provide guidance and tools to data analysts for the immediate situation with existing open data. 

Response to individual points:

1. We are not trying to create a continent-wide master facility list. We agree that an external created database is not the best approach for local acceptance. Instead we document what existing open-data sources are available. We agree that engagement with governments is to be encouraged. However, the aim of this paper is to provide a rapid picture of the current data situation. We do not have the resources to engage with the governments of 53 countries. We applaud projects (e.g. GRID3) that are engaging individual governments to improve the quality of their open data. We suggest that our complementary approach of making it easier to use existing open-data can act as an added incentive for governments to improve data provision. 

2. We agree that a standardised classification of health facility types would be useful, and we provide tools that make it easier to see the differences between countries. However (related to the previous point) we are not the right team to be imposing a classification on local data. So in this paper we deliberately didn’t want to mask national differences. We think there is considerable value in single country analyses, we are less concerned with multi-country studies.

3. We agree with the difficulties of keeping any database up-to-date. Again, we do not aim to create a database. We provide tools that can be used on different data sources as they change over time. The tools are deliberately designed to cope with changes in the data. Healthsites.io do have plans for how location data can be validated and improved through collaboration with governments. We will add text and a link to this to the paper.

4. We do not aim to create a database to inform COVID-19 response but to support the response. We aim to help improve health facility location data use now and in future by providing tools that make the data more transparent and easier to use. The same data have since been used by others to estimate access to health care for older adults in sub-Saharan Africa and the implications for the COVID-19 response (submitted and published after our submission).   

The reviewer's points highlight existing difficulties with health facility location data. We are not able to solve all of them. Nevertheless, our open-source approach aims to make existing open data more useable and provide steps to improvement.

# Peer review: Reviewer response for version 1

**DOI:** 10.21956/wellcomeopenres.17637.r39416

**Published:** 2020-07-28

**Authors:** Olusesan Ayodeji Makinde

**Affiliations:** 1 Viable Knowledge Masters, Abuja, Nigeria

Thanks for your effort in putting this manuscript together. While I like the creativity of developing an app that can identify health facilities across Africa and identify duplicates based on open access data, I fail to see how your write up links to the COVID-19 response adequately. I'd rather have you describe the app alone and its strengths than link it to the COVID-19 response especially as there is limited data to provide any meaningful support in the response. This is where the weakness I observed resides. I will advise that you work further on the manuscript by focusing on what the app can offer or to address the quality of the data available which you certainly touch on significantly. In the second suggestion, you might change the title of your manuscript to focus on 'Quality of Available Open Access Data for COVID-19 response using a novel app'. The amount of work needed to meet the second suggestion will not be too much. The current title does not convey appropriately what the article is about.

Is the work clearly and accurately presented and does it cite the current literature?

Yes

If applicable, is the statistical analysis and its interpretation appropriate?

Not applicable

Are all the source data underlying the results available to ensure full reproducibility?

Yes

Is the study design appropriate and is the work technically sound?

Partly

Are the conclusions drawn adequately supported by the results?

Partly

Are sufficient details of methods and analysis provided to allow replication by others?

Partly

Reviewer Expertise:

Epidemiology, Health Facility Assessments, Health Informatics

I confirm that I have read this submission and believe that I have an appropriate level of expertise to state that I do not consider it to be of an acceptable scientific standard, for reasons outlined above.

# Response

**Published:** 2020-10-27

**Authors:** Andy South

We appreciate the consideration of the reviewer. We had been waiting for further promised reviews but are responding now given the delay. We will amend the manuscript after they are received. 

The reviewer rightly points out that there are limitations to current open data on health facility locations and attributes in Africa that limit utility for the response to COVID-19. It is the intention of our paper to provide open-source, reproducible, methods to make the data more useable and for making these limitations clearer. We were not fully aware of the limitations before we explored the data ourselves. Current limitations in the data do not mean they are not useful at all. The same data have since been used by others to estimate access to health care for older adults in sub-Saharan Africa and the implications for the COVID-19 response (submitted and published after our submission).

We were disappointed that the review does not mention the open-source code that we provide, and describe, to allow others to progressively explore and improve the data. As suggested, we will add text describing how our tools can be used to address the data quality. These data are useful beyond the COVID-19 response. We accept current limitations with respect to COVID-19 and will make these clearer in an amended version when we have received further review. We could remove COVID-19 from the paper title.
